# New germline mutations in the hypervariable minisatellite *CEB1* in the parents of children with leukaemia

**DOI:** 10.1038/sj.bjc.6603706

**Published:** 2007-03-27

**Authors:** B G Davies, A Hussain, S M Ring, J M Birch, T O B Eden, M Reeves, Y E Dubrova, G M Taylor

**Affiliations:** 1 Cancer Immunogenetics Laboratory, Division of Human Development, University of Manchester, Manchester, UK; 2 ALSPAC DNA and Cell Line Bank, University of Bristol, Bristol, UK; 3 CRUK Paediatric and Familial Cancer Study Group, University of Manchester, Manchester, UK; 4 Academic Unit of Paediatric Oncology, Division of Human Development, University of Manchester, Manchester, UK; 5 Department of Genetics, University of Leicester, Leicester, UK

**Keywords:** childhood leukaemia, minisatellite, germline, mutation

## Abstract

Gardner and co-workers advanced the hypothesis that the Seascale leukaemia cluster could have been caused by new mutations in germ cells, induced by paternal preconceptional irradiation (PPI) exposure at the Sellafield nuclear installation. Since evidence has shown that PPI can increase the *de novo* germline mutation rate in hypervariable minisatellite loci, we investigated the hypothesis that sporadic childhood leukaemia might be associated with an increased parental germline minisatellite mutation rate. To test this hypothesis, we compared *de novo* germline mutation rates in the hypervariable minisatellite locus, *CEB1*, in family trios (both parents and their child) of children with leukaemia (*n*=135) compared with unaffected control families (*n*=124). The majority of case and control germline mutations were paternal (94%); the mean paternal germline mutation rates of children with leukaemia (0.083) and control children (0.156) were not significantly different (odds ratio, 95% confidence interval: 0.50, 0.23–1.08; *P*=0.11). There were no significant differences in case and control parental allele sizes, case and control germline mutation progenitor allele sizes (2.74 *vs* 2.54 kb; *P*=0.56), case and control mutant allele sizes (2.71 *vs* 2.67 kb; *P*=0.90), mutant allele size changes (0.13 *vs* 0.26 kb; *P*=0.10), or mutational spectra. Within the limitation of the number of families available for study, we conclude that childhood leukaemia is unlikely to be associated with increased germline minisatellite instability.

Acute leukaemia accounts for about 30% of childhood malignancies in the UK, with an average incidence rate of 4/100 000/year (Little, 1999; Stiller, 2004). Childhood leukaemia is thought to be initiated during early development by unrepaired chromosome breaks in a haemopoietic progenitor exposed to environmental carcinogens (Greaves, 1997, 1999). However, epidemiological studies have failed, with one exception, to identify decisively these carcinogens. This exception involves exposure of the unborn child to X-rays *in utero*, which is associated with a ∼50% increased risk of childhood leukaemia (Stewart *et al*, 1956; Stewart and Kneale, 1968; Stewart and Kneale, 1970; Bithell and Stewart, 1975; Doll and Wakeford, 1997; Wakeford and Little, 2003).

The discovery of an unusual cluster of childhood leukaemia cases in the village of Seascale, close to the Sellafield nuclear site in Cumbria, UK, raised concerns about fetal and neonatal exposure to environmental ionising radiation (IR; reviewed in Beral, Roman and Bobrow, 1993). To identify the cause of the Seascale cluster more precisely, a case–control study of children born in the village compared with those born elsewhere was carried out by Gardner *et al* (1990). The findings of this study were interpreted as suggesting that exposure to IR of fathers working at Sellafield might be leukaemogenic in their offspring, by a mechanism involving IR-induced *de novo* mutations in sperm during the preconceptional period. This preconceptional irradiation (PPI) hypothesis suggested a new mechanism of leukaemogenesis in humans, and was highly controversial, as there was little evidence from other sources to suggest that childhood leukaemia could be caused by germline mutation. Moreover, fathers' radiation exposure was considered to be insufficient to cause the mutation rate required for the number of cases observed (Evans, 1990; Doll *et al*, 1994; Wakeford *et al*, 1994). Additionally, there was no evidence of a germline effect among the children of Japanese atomic bomb survivors (Kodaira *et al*, 1995, 2004; Izumi *et al*, 2003). Furthermore, as radiation damage is randomly distributed across the genome, PPI should have caused an increase in single gene disorders (Doll *et al*, 1994). Although Parker *et al* (1999) reported an increased risk of stillbirth in the offspring of men working at Sellafield exposed to IR, this was not confirmed by Doyle *et al* (2000) in a study of nuclear industry workers.

Subsequent to the Gardner study, Dubrova *et al* (1996) reported that PPI caused an increased *de novo* germline mutation rate in certain human minisatellite tandem repeat DNA loci. Owing to the high spontaneous germline mutation rate of some minisatellites (>1000 × higher than most protein-coding loci), a PPI effect could be detected in a substantially smaller population than required to detect mutations in protein-coding loci (Dubrova *et al*, 1993, 1996, 1997). Studies of two different populations, one exposed to IR from radionuclide-contaminated land following the Chernobyl accident (∼0.5 Gy, Dubrova *et al*, 1996, 1997, 2002a), the other from the fallout of nuclear weapons tests (>1 Sv; Dubrova *et al*, 2002b), revealed an approximate doubling in germline minisatellite mutation rates due to PPI. These and other data suggest that minisatellite mutation rates may be useful biomarkers of germline genetic effects caused by environmental mutagens such as IR (Yauk, 2004; Bouffler *et al*, 2006). As minisatellites do not appear to be the direct target of mutagen-induced DNA damage (Dubrova *et al*, 1996, 1997), it is thought that they may act as a marker for general genome sensitivity or instability (Niwa, 2003).

On the basis of the PPI hypothesis for the aetiology of the Seascale leukaemia cluster (Gardner *et al*, 1990), and increased *de novo* germline minisatellite mutation because of PPI, we surmised that a proportion of sporadic childhood leukaemias might be associated with an increase in the rate of new germline minisatellite mutations owing to undetected exposures of parental germ cells to mutagens such as radiation. To address this, we compared germline mutation rates in the hypervariable human minisatellite, *CEB1* (Vergnaud and Denoeud, 2000) in the families of children with leukaemia (*n*=135) and control families (*n*=124), using a sensitive PCR-based minisatellite genotyping assay.

## MATERIALS AND METHODS

### Case and control samples

The case families were a cross-sectional series of children with acute leukaemia (*n*=145) treated at Royal Manchester Children's Hospital, UK, and their parents (*n*=288; two cases were monozygotic (MZ) twins). Blood samples were obtained from case children at diagnosis and in remission and from their parents. Controls were two series of family trios consisting of unaffected children (*n*=124) and their parents (*n*=248). One control series consisted of buccal scrape samples from children (*n*=64) and blood samples from their parents (*n*=128), recruited as part of the UK Childhood Cancer Study (2000) in the North West (NW) of England. The second control series consisted of blood samples from children (*n*=60) obtained as part of the Avon Longitudinal Study of Parents and Children (ALSPAC; Jones *et al*, 2000) and buccal samples from their parents (*n*=120; Ring *et al*, 2001). Sample collection and analyses were carried out with the approval of Local Research Ethics Committees.

## GENOMIC DNA EXTRACTION

Genomic DNA (gDNA) was extracted from case and control blood samples using phenol/chloroform or Nucleon BACC2 genomic DNA extraction kits (Amersham Biosciences, Chalfont St Giles, UK). gDNA was extracted from buccal samples using MasterAmp™ Buccal Swab DNA Extraction Kits (Epicentre Technologies, Madison, USA). DNA concentrations were measured by fluorimetry on a TBS-380 Mini-Fluorometer (Turner BioSystems Inc., Sunnyvale, CA, USA) using a PicoGreen® (Molecular Probes, Paisley, UK) based assay.

### *CEB1* primer sequences and PCR amplification conditions

*CEB1* alleles were amplified by PCR using the 3′ *CEB1* primer P14 (5′-ggatcctctcctgtgcctttcct-3′) described by Buard *et al* (1998) and a 5′ primer, MAR1 (5′-gaattttcagtgagagtcggcc-3′). MAR1 was designed for this study to avoid single nucleotide polymorphisms (SNPs) flanking the *CEB1* minisatellite, using the *CEB1* sequence (accession no. AF048727) and SNP information kindly provided by Dr Jerome Buard (personal communication). Polymerase chain reactions were performed on an MJ Research DNA Engine (Waltham, MA, USA) in 50 *μ*l reaction mixtures consisting of 1 *μ*l of gDNA (50 ng) and 0.5 *μ*l (2.5 units) of platinum *Taq* DNA polymerase high fidelity (Invitrogen, Paisley, UK) added to 48.5 *μ*l of Mg^2+^-titrated PCR master-mix. The PCR master-mix (19.4 ml) was prepared with the following final reagent concentrations: 0.5 *μ*M each high-performance liquid chromatography-purified *CEB1* primer (Eurogentec, Southampton, UK), 0.7 mM MgSO_4_ (50 mM MgSO_4_ with platinum *Taq* DNA polymerase), 0.2 mM dNTPs (PCR nucleotide mix, Amersham Biosciences) 5% (v/v) DMSO (Sigma, , Dorset, UK), 0.2 mg ml^−1^ bovine serum albumin (ultra pure, non-acetylated; Ambion, Warrington, UK), and 1 × high-fidelity PCR buffer (50 × high-fidelity PCR buffer with platinum *Taq* DNA polymerase HF). Amplification conditions were 94°C for 1 min 30 s, 30 cycles at 94°C for 15 s, 60°C for 30 s, 70°C for 10 min, and a final extension step of 70°C for 15 min.

### Electrophoresis

Polymerase chain reaction products containing *CEB1* alleles amplified from the gDNA of family trios (father, mother, and child) were loaded onto a 40-cm long 0.7% (w/v) agarose gel (SeaKem LE Agarose, Cambrex BioScience, East Rutherford, NJ, USA). Gels were run at 2 V cm^−1^ in 1 × TBE buffer (Crystal Buffers, Severn Biotech, Kidderminster, UK) for approximately 23 h, until the 400 bp marker from the DNA ladder (1 kb plus DNA Ladder, Invitrogen) was about to run off the gel. Few alleles <400 bp were observed, but when present were sized by a shorter gel run. Multiple ladder lanes were included across each gel to enable the image analysis software to calculate the gel retardation factor (*R*_f_ value) for any region of the gel, allowing more accurate allele sizing. Gels were stained with SYBR gold nucleic acid gel stain (Molecular Probes) in 1 × TBE buffer (pH 8.0), the bands being visualised by UV transillumination on a Gel Doc 2000 instrument (Bio-Rad, Hemel Hempstead, UK). The captured gel image in TIFF file format was set up with an image resolution of 38.4 pixels cm^−1^.

### Identification of *CEB1* alleles and mutations

Allele typing and mutation detection were performed on the gel image using Phoretix™ 1D Advance software (Nonlinear Dynamics, Newcastle upon Tyne, UK). Offspring bands (alleles) were deemed to be germline mutations if no corresponding alleles were detected in either parent. Band shifts were measured in bps so that allele sizes could be adjusted for variations in *R*_f_. This was carried out by placing a simulated band on the gel image at a position 1.5 mm (corresponding to ∼8 pixels) from the band of interest, in the direction of the progenitor band in the parent. The progenitor band was taken to be the parental allele closest in size to the mutant band (Dubrova *et al*, 1996). If the size of the putative mutant allele was equal to or larger (for an expansion), or equal to or smaller (for a deletion), than the simulated band (as measured in bp), it was classified as a mutation. All mutations detected were verified by repeat analysis, in which gDNA was re-amplified and run on a second gel.

### Verification of parentage

Biological parentage was verified in case and control families from NW England with suspected germline mutations using three short tandem repeat (STR) markers (*VWA*, *TH01*, and *FGA*) from the FBI combined DNA index system (CODIS) and the minisatellite, *D1S80*, as described in the National Institute of Standards and Technology STR DNA internet database (http://www.cstl.nist.gov/biotech/strbase/; Ruitberg *et al*, 2001) and using HLA-DPB1 typing data already available for families (Taylor *et al*, 2002). Primer sequences for STRs were: *VWA* 5′-ccctagtggatgataagaataatcagtatg-3′ and 5′-ggacagatgataaatacataggatggatgg-3′; *TH01* 5′-attcaaagggtatctgggctctgg-3′, 5′-gtgggctgaaaagctcccgattat-3′; and *FGA* 5′-attatccaaaagtcaaatgccccatagg-3′, 5′-atcgaaaatatggttattgaagtagctg-3′. Primer sequences for *D1S80* were: 5′-gtcttgttggagatgcacgtgccccttgc-3′, 5′-gaaactggcctccaaacactgcccgccg-3′ (Kasai *et al*, 1990). High-performance liquid chromatography-purified primers were obtained from Eurogentec or Applied Biosystems (Warrington, UK), the forward primer for each set being labelled at the 5′-end with VIC (*VWA*), NED (*TH01*), or 6-FAM (*FGA* and *D1S80).* All four loci were co-amplified and analysed on an ABI-310 genetic analyser (Applied Biosystems). Reagent concentrations in 20 *μ*l reaction volumes were 1 × ReddyMix™ PCR master mix with 2.5 mM MgCl_2_ (ABgene, Epsom, UK), 0.25 *μ*M each primer and 2.5 ng *μ*l^−1^ DNA. Polymerase chain reaction amplification conditions were 95°C for 2 min, 30 cycles at 95°C for 30 s, 63°C for 30 s, 70°C for 2 min, and a final extension step of 70°C for 5 min. Parentage was verified in mutation carriers if alleles in at least two of the four tandem-repeat loci were present in parents; DP types were used as an independent check. Parentage of all ALSPAC control family trios was verified using four STR loci (*D5S818*, *D7S820*, *D13S317*, *D16S539*; http://www.cstl.nist.gov/biotech/strbase/) by multiplex PCR using GammaSTR kits (Promega, Southampton, UK) as described by Ong *et al* (2004).

### Data analysis

Case–control *CEB1* germline mutation rates were compared by calculating cross-product odds ratios (ORs) and 95% confidence intervals (CI) using the RERI program from the linkage utility package, LINKUTIL, by the Sheehe method (http://linkage.rockefeller.edu/soft/linkutil/). The 2 × 2 programme in LINKUTIL was used to determine *P* values for case–control differences using Fisher's exact test. Mean case–control parental ages and germline progenitor allele sizes were compared using unpaired *t*-tests by Minitab release 13.30. Case–control parental allele size distributions were compared using the Kolmogorov–Smirnov Z two-independent samples test. Statistical power was calculated using nQuery Advisor v2.0 (Statistical Solutions, Cork, Ireland).

## RESULTS

### Case–control characteristics

A total of 145 leukaemia cases from 144 families were originally included in the study. Two cases with B-cell precursor ALL (BCP ALL) were MZ twins and were treated as one case in the analysis. Control families consisted of 124 unaffected children and their parents. Nine case families were excluded from the study; five because of failure to detect *CEB1* alleles; four because of non-parentage, leaving 135 informative case families. Table 1 shows the number of informative case children with each leukaemia subtype. B-cell precursor ALL is the predominant subtype (70%), the majority (68%) of case samples were collected at diagnosis. The mean age of cases at diagnosis of leukaemia was 5 years 5 months (range: 0.2–15.0 years). The mean paternal age at the birth of case children was 30.4±(s.d.) 5.4 years; for the control series were the following, NW: 31.0±6.3 years; ALSPAC: 32±5 years. Mean maternal ages were: case children 28.5±5.7 years; NW controls: 29.1±4.6 years; ALSPAC controls: 30±4.1 years. There were no significant differences between case and control parental ages (*P*>0.05)

### Amplification and genotyping of *CEB1* alleles

**Figure 1:**
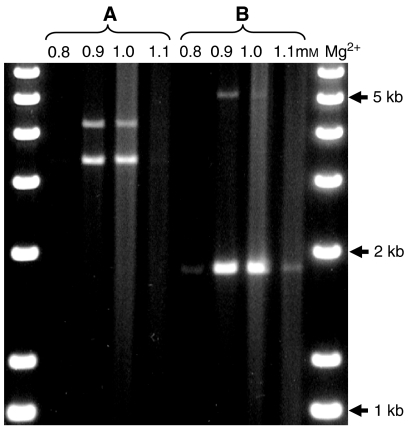
Effect of Mg^2+^ concentration on amplification of *CEB1* alleles in two gDNA samples (**A** and **B**) by PCR. No alleles were resolved at 0.8 or 1.1 mM Mg^2+^, whereas alleles were optimally detected at 0.9–1.0 mM.

*CEB1* genotyping was carried out by amplifying gDNA in PCR to levels visible in SYBR gold-stained gels. A very precise Mg^2+^ concentration (to within 0.1 mM) was found to be required for successful amplification and allele discrimination (Figure 1). Using this method, we obtained an allele amplification efficiency of 90%, with no amplification artifacts and consistent results in repeat sample analysis.

### Case–control germline mutation rate comparison

**Figure 2:**
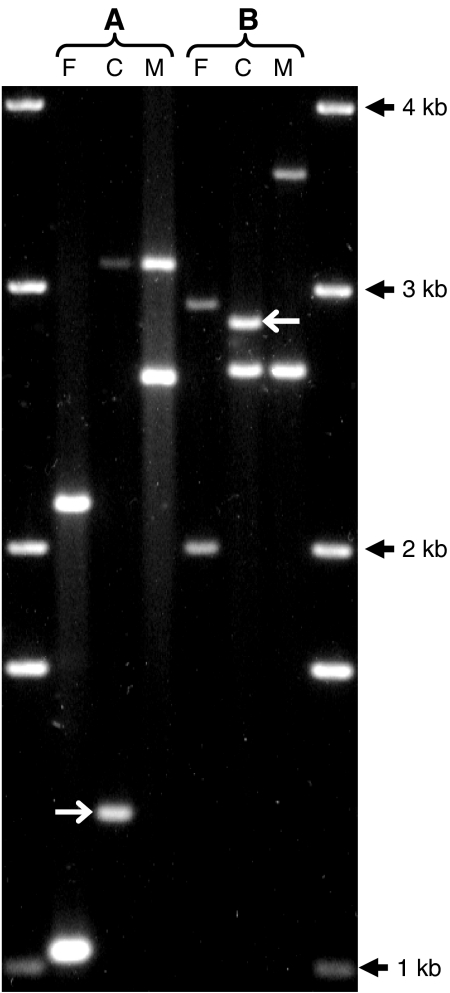
Detection of *de novo* germline *CEB1* mutations in two family trios (**A** and **B**) following PCR and gel electrophoresis. Mutant alleles are indicated by white arrows. Family A has a paternal mutation corresponding to a seven repeat expansion. Family B has a paternal mutation corresponding to a one repeat deletion. To ensure accurate band comparison between adjacent lanes samples were loaded in the order father (F), child (C), mother (M).

**Figure 3:**
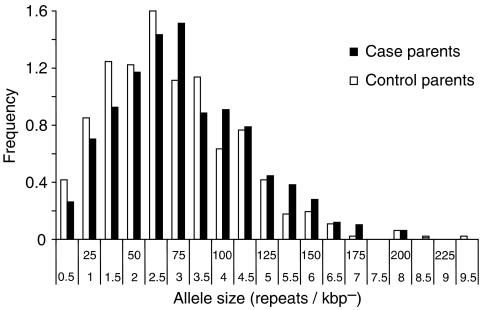
Size distribution of *CEB1* alleles in the parents of case and control children (number of alleles: case, *n*=496; control, *n*=457). DNA fragments were grouped into 0.5 kb intervals from 0.5 to 9.5 kb, with the number of repeat units shown, where one consensus repeat=40 bp). Differences between case and control parent allele distributions were not significant in the Kolmogorov–Smirnov two-sample test (*P*>0.05).

Germline mutations were detected in 11 of 135 case and 20 of 124 control children. Representative examples of mutations (one expansion and one deletion) in two case families are shown in Figure 2. All case mutations were paternal in origin; two of 21 control mutations were maternal. One control but no cases had a double, paternal and maternal, mutation. Table 2 shows that there is no significant difference in parental mutation rates in case (0.041) and control (0.086) families (OR, 95% CI: 0.47, 0.23–0.98; *P*=0.061). Paternal mutation rates in case (0.083) and control families (0.156) were also not significantly different (OR, 95% CI: 0.50, 0.23–1.08; *P*=0.11). Analysis of mutation rates in relation to leukaemia subtype (Table 3) shows no significant case–control differences, though the small number of pro B and T ALL and acute myeloid leukaemia (AML) suggests that these results should be treated with caution. The subtype with the highest mutation rate was T ALL (0.111), but this was not significantly different from controls (OR, 95% CI: 0.8, 0.22–3.00; *P*=0.93). The mean age of case fathers with a germline mutation (29.9 years) was not significantly different from NW control fathers with a mutation (33.9 years; *P*=0.27). The mean age of case fathers whose children had a germline mutation (29.9 years) was not significantly different from fathers of case children who had no mutation (30.4 years; *P*=0.81). The mean germline mutation rate in diagnostic samples (0.039) was not significantly different from remission samples (0.046; *P*>0.05). The *CEB1* allele size distributions (Figure 3) in case parents (mean allele size: 2865 bp; 72 repeats) was not significantly different from control parents (mean allele size: 2360 bp; 59 repeats; *P*>0.05).

### Germline mutation spectrum

**Figure 4:**
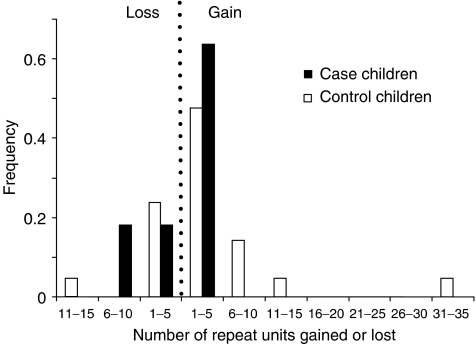
Frequency distribution of germline CEB1 mutations in case and control children by size and polarity, with mutations grouped into repeat unit changes of up to five units. In leukaemia case children, all gains are 1-5 repeats and losses 1-10 repeats, whereas in control children gains are 1-35 repeats and losses 1-15 repeats.

In cases, there were seven (64%) mutant alleles with an increased and four with a decreased number of repeats, whereas in the controls, 15 (71%) mutants increased and six decreased in size. The size of the mutational events was similar in the cases and controls. The mean mutant allele size in cases (2.71 kb) was not significantly different from controls (2.67 kb; *P*<0.90). There was no difference between mean progenitor allele size in cases (2.74 kb) and controls (2.54 kb; *P*=0.56). Most mutations (75%) involved the gain or loss of 1–5 repeat units (Figure 4), but there was no significant difference in the size change in cases (0.13 kb) and controls (0.25 kb; *P*=0.10). The largest control mutation was an expansion of 32 repeat units (progenitor allele size: 2.09 kb); the largest case mutation was a deletion of nine repeats (progenitor allele size: 2.55 kb) in a child with BCP ALL.

## DISCUSSION

This study was designed to address the hypothesis that sporadic childhood leukaemia is associated with an increased rate of *de novo* germline mutation in the hypervariable minisatellite, *CEB1*. The results show, with some qualifications, that there is no association. The hypothesis is based on the notion that exposure of parental germ cells to mutagenic agents such as IR can lead to an increased risk of childhood leukaemia, the PPI hypothesis (Gardner *et al*, 1990). Here, we used minisatellite mutation as a biomarker of *de novo* germline leukaemogenic damage. We found no evidence for quantitative (germline mutation rate) or qualitative (mutation spectrum) differences in *CEB1* minisatellite mutations between case and control families. As far as we are aware no previous study of this type has been reported in childhood leukaemia.

Unlike other studies of germline minisatellite mutation rate in relation to parental IR exposure (reviewed by Bouffler *et al*, 2006), no parental radiation dosimetry data were available for our leukaemia cases. Accurate environmental radiation and mutagen exposure data are extremely difficult to collect in a non-occupational setting, but it is safe to assume in these cases that environmental radiation exposures were extremely low. The catchment area for our sporadic cases covers an area of NW England south of Cumbria, where levels of occupational or environmental radiation are far below those to which the Sellafield workers were exposed. As *CEB1* exhibits high rates of spontaneous and induced mutation, our study should have revealed evidence of undetected parental mutagen exposure associated with childhood leukaemia. From our prior hypothesis of an increased case *de novo* germline mutation rate, our study of 135 informative case and 124 control families has 90% power to detect a significant doubling (one-sided, *P*=0.05) of the control paternal mutation rate in cases (i.e., from 0.156 in controls to 0.312 in cases). Although we analysed only one minisatellite locus, in contrast to others using up to eight loci (reviewed by Bouffler *et al*, 2006), it is worth noting that significance in multiple locus tests requires correction for the number of loci tested, whereas our result does not.

Germline minisatellite mutation analysis has routinely been carried out using Southern blotting and probing of PCR-amplified gDNA (Dubrova *et al*, 1996; Stead and Jeffreys, 2000). However, this is expensive on gDNA, leading to difficulties in testing children with haemopoietic malignancies where numbers of peripheral blood leucocytes may be reduced by disease or therapy. Using a PCR-based genotyping assay that directly visualised *CEB1* alleles on long-read agarose gels using a UV transilluminator, direct analysis of PCR products has provided a robust alternative, allowing fine resolution and sizing of mutations. Careful titration of Mg^2+^ gave reproducible amplification of *CEB1* alleles to visible levels, which we found useful for the genotyping of other PCR-able minisatellite loci such as *B6.7* (Tamaki *et al*, 1999). Several other hypervariable minisatellites (Vergnaud and Denoeud, 2000) should be amenable to analysis using this PCR-genotyping assay.

The majority of *de novo* germline mutations detected in the case and control series in the present study (30/32) were paternal in origin, a result previously reported for *CEB1* (Vergnaud *et al*, 1991). We examined the size change and polarity of mutational events (increase or decrease in allele size), but found no difference between the case and control series. In both groups, allele size expansions were more prevalent than deletions (with an overall ratio of 2.2 : 1 in favour of expansion) and a large proportion of mutations involved the gain or loss of only 1–3 repeat units (73 cases and 62% controls). Small mutations such as these are reminiscent of the stepwise model for microsatellite expansion (Nag, 2003) though the evidence suggests that virtually all *CEB1* germline mutations are initiated by recombinogenic events occurring during gametogenesis (Buard *et al*, 1998, 2000) and not as a result of DNA replication slippage.

In view of the tendency of some single gene disorders to increase in frequency with paternal age, we compared paternal ages at the birth of case and control children with germline mutations. There was no evidence that fathers transmitting germline mutations were significantly older than those who did not. Although mean allele sizes differed slightly between cases and controls (72 and 59 repeats, respectively), there was no significant difference between the allele size distributions suggesting that paternal allele size is not a factor in the risk of leukaemia. This was confirmed by the finding that the sizes of the germline mutation progenitor alleles in cases and controls were not significantly different. The analysis of *CEB1* mutation rate in relation to leukaemia subtype was limited by the small number of cases with progenitor B-cell ALL, T ALL, and AML. However, our preliminary finding suggests that none of the leukaemia subtypes had a mutation rate that was higher than the controls.

The type of sample used in the *CEB1* genotyping was a potential confounder. We collected 69% of case samples at diagnosis and 31% in remission. The diagnostic samples contained varying percentages of leukaemic blasts (1–96%), which could have influenced the result if the blasts contained *CEB1* somatic mutations. However, comparison of the mutation frequency in the diagnostic (seven mutations in 93 children (7.5%)) and remission samples (four mutations in 42 children (9.5%)) suggests little difference. In seven case families, where paired diagnostic and remission samples were available for analysis, we found no differences in *CEB1* alleles in the paired samples, even though three of the diagnostic samples had >73% blasts. In the control families, buccal instead of blood samples were obtained from children in the NW series and from parents in the ALSPAC series. The overall germline mutation rate in the two series was little different (0.0833 (NW) and 0.0877 (ALSPAC)), confirming preliminary studies showing that the genotypes of buccal samples do not differ from blood. The *CEB1* somatic mutation rate is known to be very low (Vergnaud *et al*, 1991; Buard *et al*, 1998; 2000). Buard *et al* (2000) reported that the *CEB1* somatic mutation rate in blood was 1.8 × 10^−4^ as compared with a ∼6% germline mutation rate in the same donor.

In summary, our results show, within the limitations of the numbers of case and control families available for study, no evidence of an increased rate of *de novo CEB1* germline minisatellite mutation in the parents of children with sporadic leukaemia. Our provisional conclusion is that new germline minisatellite mutations, used as a surrogate measure of parental mutagen exposure on germ cell stability, are unlikely to be a major factor in the aetiology of childhood leukaemia. Although our data do not rule out the possibility that prezygotic mutagenesis could account for rarer subtypes of childhood leukaemia, approaches to this question based on population case–control comparisons are unlikely to have sufficient power to provide a definitive result. The report by Dickinson and Parker (2002) confirming the original findings of Gardner *et al* (1990) of a statistical association between a father's radiation dose at Sellafield and the child's risk of developing leukaemia suggests that future studies of germline minisatellite mutations in childhood leukaemia should focus specifically on case clusters. Thus, the collection of DNA from family trios in such clusters (Steinberg *et al*, 2006) has a high priority.

## Figures and Tables

**Table 1:** Details of leukaemia case series in this study

	**Number of samples obtained at**		
**Leukaemia cases**	**Diagnosis**	**Remission**	**Total**
BCP ALL	66	29	95
Pro-B ALL	6	2	8
T ALL	10	9	19
AML^a^	11	2	13
Total cases	93	42	135

AML, acute myeloid leukaemia; BCP ALL, B-cell precursor ALL; Pro-B ALL, progenitor B-cell ALL; T ALL, T-cell ALL.

a One case of chronic myeloid leukaemia and one case of myelodysplastic syndrome.

**Table 2:** Germline *CEB1* mutation rate in parents of childhood leukaemia cases and controls

**Comparison**	**Cases**	**Controls**	**OR**	**95% CI**	Two-sided P^*^
Informative family trios (*n*)	135	124			
Informative offspring (*n*)	136	124			
Paternal alleles (*n*)	132	122			
Maternal alleles (*n*)	131	122			
Paternal germline mutations	11	19			
Maternal germline mutations	0	2			
Paternal mutation rate	0.083 (8.3%)	0.156 (15.6%)	0.50	0.23–1.08	0.11
Maternal mutation rate	0	0.016 (1.6%)	0.18	0.02–1.79	0.463
Parental mutation rate	0.041 (4.1%)	0.086 (8.6%)	0.47	0.23–0.98	0.061

CI, confidence interval; *n*, number; OR, odds ratio.

^*^Two-sided *P* value in Fisher's exact test.

**Table 3:** Paternal *CEB1* germline mutation rate in relation to childhood leukaemia subtype

**Leukaemia subtype**	**Paternal alleles**	**Paternal mutations**	**Paternal mutation rate**	**OR**	**95% CI**	**Two-sided P**
BCP ALL	93	9	0.097 (9.7%)	0.60	0.27–1.34	0.28
Pro-B ALL	8	0	0	0.31	0.04–2.60	0.54
T ALL	18	2	0.111 (11.1%)	0.80	0.22–0.46	0.93
AML	13	0	0	—	—	—
Total cases	132	11	0.083 (8.3%)	0.50	0.23–1.08	0.11
Controls	122	19	0.156 (15.6%)			

AML, acute myeloid leukaemia; BCP ALL; B-cell precursor ALL; Pro-B ALL, progenitor B-cell ALL; T ALL, T-cell ALL.

^a^Leukaemia subtype in relation to paternal allele mutation rate.
